# Significance of CD133 positive cells in four novel HPV-16 positive cervical cancer-derived cell lines and biopsies of invasive cervical cancer

**DOI:** 10.1186/s12885-018-4237-5

**Published:** 2018-04-02

**Authors:** Shifa Javed, Bal Krishan Sharma, Swati Sood, Sanjeev Sharma, Rashmi Bagga, Shalmoli Bhattacharyya, Charan Singh Rayat, Lakhbir Dhaliwal, Radhika Srinivasan

**Affiliations:** 1 Molecular Pathology Laboratory, Department of Cytology and Gynecological Pathology, Postgraduate Institute of Medical Education and Research, Chandigarh, PIN-160012 India; 2 Department of Obstetrics and Gynecology, Postgraduate Institute of Medical Education and Research, Chandigarh, India; 3 Department of Histopathology, Postgraduate Institute of Medical Education and Research, Chandigarh, India; 4 Department of Biophysics, Postgraduate Institute of Medical Education and Research, Chandigarh, India

**Keywords:** CD133, Cancer stem cells, Cervical cancer, HPV16, Cell lines, Low passage

## Abstract

**Background:**

Cervical cancer is a major cause of cancer-related mortality in women in the developing world. Cancer Stem cells (CSC) have been implicated in treatment resistance and metastases development; hence understanding their significance is important.

**Methods:**

Primary culture from tissue biopsies of invasive cervical cancer and serial passaging was performed for establishing cell lines. Variable Number Tandem Repeat (VNTR) assay was performed for comparison of cell lines with their parental tissue. Tumorsphere and Aldefluor assays enabled isolation of cancer stem cells (CSC); immunofluorescence and flow cytometry were performed for their surface phenotypic expression in cell lines and in 28 tissue samples. Quantitative real-time PCR for stemness and epithelial-mesenchymal transition (EMT) markers, MTT cytotoxicity assay, cell cycle analysis and cell kinetic studies were performed.

**Results:**

Four low-passage novel cell lines designated RSBS-9, − 14 and − 23 from squamous cell carcinoma and RSBS-43 from adenocarcinoma of the uterine cervix were established. All were HPV16+. VNTR assay confirmed their uniqueness and derivation from respective parental tissue. CSC isolated from these cell lines showed CD133^+^ phenotype. In tissue samples of untreated invasive cervical cancer, CD133^+^ CSCs ranged from 1.3–23% of the total population which increased 2.8-fold in radiation-resistant cases. Comparison of CD133^+^ with CD133^−^ bulk population cells revealed increased tumorsphere formation and upregulation of stemness and epithelial-mesenchymal transition (EMT) markers with no significant difference in cisplatin sensitivity.

**Conclusion:**

Low-passage cell lines developed would serve as models for studying tumor biology. Cancer Stem Cells in cervical cancer display CD133^+^ phenotype and are increased in relapsed cases and hence should be targeted for achieving remission.

**Electronic supplementary material:**

The online version of this article (10.1186/s12885-018-4237-5) contains supplementary material, which is available to authorized users.

## Background

Cancer of cervix is the fourth most common cancer among women and the third most common cause of cancer related mortality worldwide [1]. Developing countries contribute to nearly 80% of cervical cancer cases with an incidence to mortality ratio of nearly 50% resulting in a large number of cancer related deaths [1] Advanced stage at presentation and resistance to standard chemoradiation are important factors leading to a high mortality and in turn may be attributed to residing cancer stem cells [CSC] which, by definition are a self-renewing population of cells within a tumor capable of initiation and maintenance of a tumor. A consensus of five defining criteria are required to prove the existence of CSCs [2]. The expression of distinctive cell surface markers is one criterion which permits their consistent isolation, characterization and enables the exploration of alternative therapeutic strategies to target CSC [3].

CSC have been demonstrated in several solid cancers. In cancer cervix, cancer stem-like cells were first identified to exist by Feng et al. [4] and subsequently by others [5–8]. Identification of their surface phenotype is the initial step towards their isolation and enrichment for subsequent biological studies. Unfortunately, there has been no consistency in the previous reports and a consensus marker of CSC in cervical cancer is still elusive. Thus, breast cancer-resistance protein (Brcp1) [5], aldehyde dehydrogenase (ALDH^+^) [5, 6], Annexin II [6], CD44+/CK17+ [4] and CD49f [8] have all been proposed as makers for CSC based on different approaches. Moreover, only a single study by Feng et al. [4] is based on primary cultures from patient samples whereas the rest are based on highly passaged established cell lines such as HeLa, SiHa, CaSki and C33A [5–8]. Hence our initial step was to generate short-term primary cultures from tissue biopsies of invasive cervical cancer. In this process, we were able to establish 4 low-passage cell lines, all from Indian ethnicity. Further studies on the isolation and characterization of CSC were performed on these cell lines from invasive cervical carcinoma. We applied two assays that permit enrichment of CSCs, namely, the tumorsphere assay and second, a functional assay based on aldehyde dehydrogenase [ALDH] activity. Using flow cytometry and immunofluorescence studies, we established that CD133 is a surface phenotypic marker of cancer stem cells in cervical carcinoma of both the squamous cell carcinoma and adenocarcinoma subtypes. The clinical significance of the CSCs was evaluated in biopsies from 29 cases of invasive cervical cancer.

## Methods

### Patients and samples

The study was approved by the Institute’s Ethics Committee (vide letter No.IC-CCRT/07/DTM-3226 dated 16.11.2009). All patients were enrolled after obtaining a voluntary written informed consent and the study was conducted as per the Helsinki declaration (2000). Tissue samples obtained from untreated patients of invasive cervical cancer comprising of 3 radical hysterectomies in FIGO stage IIA and 30 cervical biopsies from FIGO stage IIb/III were subjected to primary culture. The sample was collected directly in Dulbecco’s Modified Eagle Medium/ Nutrient Mixture F-12 Ham (DMEM/ F12, 1:1 mixture) (Invitrogen). The rest of the specimen was subjected to formalin fixation and paraffin-embedded sections were evaluated light microscopically for histopathological diagnosis.

### Primary culture

The specimen collected was immediately mechanically disaggregated with a sterile surgical blade, washed 3 times with sterile phosphate buffered saline (PBS), digested by collagenase II by incubation for 1 h at 37 °C (Sigma-Aldrich Corp., St Louis, MO, USA), filtered through a 70 μm mesh to get rid of stromal fragments and obtain a cell suspension. The tumour cells were placed in 6 well plates (Corning) containing DMEM/ F12 supplemented with 10% fetal bovine serum (FBS, Sigma), penicillin (100 U/mL), and streptomycin (100 μg/mL) and placed in a humidified incubator at 37 °C containing 5% carbon dioxide (CO_2_) and 95% air. 17 cases showed contamination and in 16 cases successful primary cultures could be established. In 7 of these 16 cases, long-term primary cultures got established; and 4 were pursued further. The morphology of the adherent cells was observed under an indirect microscope by phase-contrast and morphology documented.

### Electron microscopy

Approximately 5 × 10^5^ cells from RSBS-14 and RSBS-43 cell lines were centrifuged gently and fixed in 3% glutaraldehyde followed by fixation in 1% Osmium tetroxide, processed and embedded in Taab-812 embedding medium. 60 nm thick sections on Nickel grids were stained with uranyl acetate and lead citrate and examined using JEOL Transmission Electron Microscope, JEM-1400Plus (JEOL, Tokyo, Japan) equipped with XR81M-B Camera (Advanced Microscopy Techniques Corp, Woburn, MA, United States).

### Karyotyping

Karyotyping was performed on metaphase spreads of RSBS-9, RSBS-14, RSBS-23 and RSBS-43 cells at passage 32 by standard G-banding.

### Tumorsphere assay

Cultured cells were also placed under anchorage-independent/stem-cell conditions in Ultra Low Attachment plates (Corning, USA) in serum-free DMEM/F12 supplemented with 5 μg/mL insulin (Sigma), 20 ng/mL human recombinant epidermal growth factor (EGF; Invitrogen), 10 ng/mL basic fibroblast growth factor (bFGF; Invitrogen), and 0.4% bovine serum albumin (BSA; Sigma). Floating aggregates or tumorspheres were observed microscopically and their numbers per well documented. Efficiency of tumorsphere formation ability was calculated based on manual counting of number of tumorspheres after plating equal numbers of sorted cells wherever required.

### ALDEFLUOR assay

Adherent cells derived from samples were used for ALDEFLUOR assay using ALDEFLUOR kit (Stem cell Technologies) as per manufacturer’s instructions detailed in Additional file 1. Based on their ALDH activity, cells were sorted as ALDH^high^ and ALDH^low^ cells (Additional file 1).

### Detection of putative markers of cancer stem cells by flow cytometry and FACS

At least 1 × 10^6^ cells derived from tumorspheres and corresponding adherent cells were screened for surface markers by flow Cytometry using fluorescent (FITC, PE, APC, Per-CP) labelled antibodies against CD117, CD90, CD44, CD49f and CD71. Isolation of the CD133^+^ cells was performed using a FACS Aria flow cytometer (BD Biosciences, USA) and analyzed further (Additional file 1).

### Direct immunofluorescence

Direct immunofluorescence was carried out on adherent cells and tumorspheres with FITC/PE labelled antibodies against CD44, CD49f and CD133 and on corresponding frozen tissue sample of cervical cancer (Additional file 1). Nuclei were counter stained with DAPI. Florescence microscopy was carried out with EVOS FL Auto cell imaging system (Invitrogen, Thermo Fisher Scientific, USA).

### Screening of patient samples of invasive cervical carcinoma for cancer stem cell percentage and patients after radiotherapy

Flow cytometric immunophenotyping (FCI) was performed on the biopsies of patients with untreated invasive cervical cancer (*n* = 22) and in 6 patients who had been treated with chemoradiation therapy 46 Gy/23 fraction external beam radiation administered concurrently with 40 mg/m^2^ cisplatin weekly dose followed by intracavitary brachytherapy and who had subsequently relapsed. The levels of CD133, CD49f and CD44 was evaluated in each case after gating for CD45 negative cells (Additional file 1).

### Human papillomavirus (HPV) screening

All cell lines developed were evaluated for HPV DNA type by PCR with type specific primers against HPV-16, − 18, − 31 and − 45 (Additional file 1: Table S1).

### Assay of growth characteristics

#### MTT assay

Cells from all the 4 cell lines generated and which were at the same passage 35 (P35) were used to plot the growth curve by using the MTT 3-(4,5-dimethylthiazol-2-yl)-2,5-diphenyl tetrazolium bromide assay detailed in Additional file 1. The growth curves were plotted and the population doubling time of these 4 cell lines were calculated during the exponential growth phase of the cells.

#### Cell cycle analysis

Cell cycle analysis was performed after labelling the cellular deoxyribonucleic acid (DNA) with propidium iodide (PI) (Invitrogen, Carlsbad, CA, USA). The cells were then subjected to cell cycle analysis by FACS (BD FACS Aria II). Each experiment was analysed in duplicate and four independent experiments were performed (see Additional file 1).

#### Variable nucleotide tandem repeat [VNTR] assay

Highly polymorphic VNTR loci D1S80 and IGH (VNTR at the immunoglobin heavy chain enhancer HSS 1.2) were used to prove and validate that the cervical cancer cell lines were derived from primary cervical cancer biopsies. Amplification of VNTR loci D1S80 (range of amplification product 387 to 723 bps) was achieved using the primers (Additional file 1).

#### Chemosensitivity assay

Cell lines RSBS-9, RSBS-14, RSBS-23 and RSBS-43 were treated with different doses of cisplatin (10, 20, 30, and 40 μM) for variable duration (24, 48, 72 h). Cytotoxicity was determined by MTT assay All experiments were performed twice in triplicates to determine the IC^50^ values.

### Quantitative real time PCR (qRT-PCR) for stemness/EMT/markers

Total RNA from the cells was isolated using the RNA isolation kit (Agilent technologies, USA) per the manufacturer’s instructions. 1 μg RNA was reverse-transcribed into cDNA using specific primers and per the manufacturer’s instructions (Thermo Fisher Scientific, USA). 1 μL of cDNA was used as template for qRT-PCR for *OCT-4, SNAIL, SLUG, VIMENTIN, ABCG2, N-CDHERIN, NANOG, TIWST* and *E-CADHERIN* using gene specific primers (Additional file 1: Table S2), and normalized to β-ACTIN housekeeping gene transcript.

## Results

### Establishment of primary cultures and permanent cell lines

Successful long-term primary cultures could be established in 7/33 or 21.2% cases; four of these were pursued and 4 permanent cell lines were derived. They were designated as RSBS-9, RSBS-14, RSBS-23 and RSBS-43 respectively with the ages of the patients being 49, 34, 45 years and 63 years respectively. All 4 cell lines were derived from cervical biopsy specimen and from patients with FIGO stage III disease. All the cell lines established were regularly checked for mycoplasma contamination.

### Morphology, ultrastructure and karyotyping of derived cell lines

**Fig. 1:**
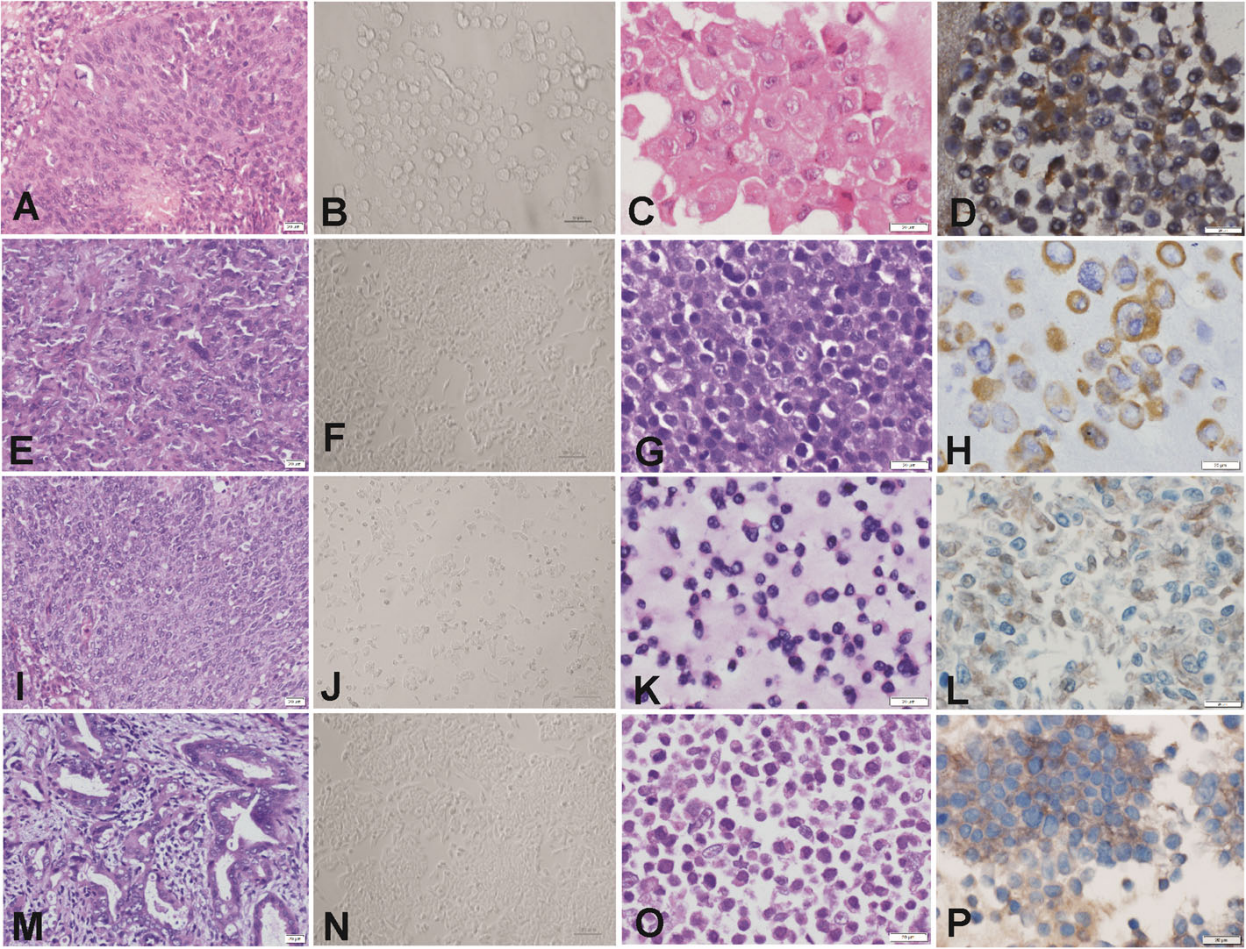
Image panel of the four novel cell lines. First column shows histology of the parental tissue and second column shows phase contrast micrograph of the cell line developed, third column shows the corresponding cell block histology and fourth column, cytokeratin positivity on immunohistochemistry. **a**-**d** RSBS-9: Keratinizing squamous cell carcinoma, moderately differentiated; **e**-**h** RSBS-14: Non-Keratinizing squamous cell carcinoma, moderately differentiated; **i**-**l** RSBS-23: Non-Keratinizing squamous cell carcinoma, poorly differentiated; and **m**-**p** RSBS-43: Adenocarcinoma

The histology of the primary tumour corresponding to the RSBS-9 cell line was a moderately differentiated keratinizing squamous cell carcinoma, for RSBS-14 and RSBS-23 cell lines were non-keratinizing squamous cell carcinoma, moderately and poorly differentiated respectively. RSBS-43 cell line was derived from a moderately differentiated adenocarcinoma. The parental tissue biopsies and the respective adherent cell lines derived are shown in Fig. 1 panel. All four cell lines grew in adherent monolayers with pavement-like epithelial morphology which exhibited contact inhibition. Immunocytochemistry on cell blocks of these adherent cell lines and showed positivity for epithelial membrane antigen and pan cytokeratin confirming their epithelial nature [Fig. 1].

**Fig. 2:**
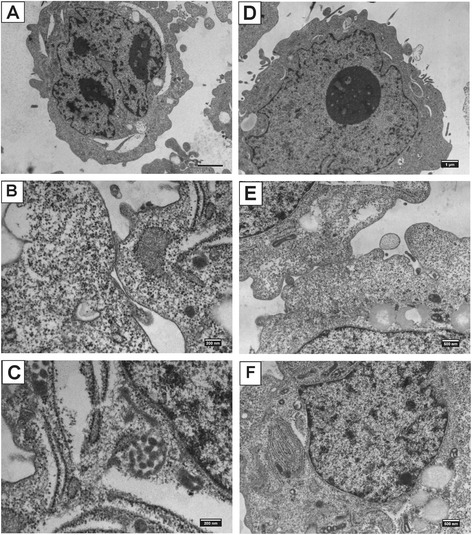
Representative images of electron microscopy of RSBS-14 (**a**-**c**) and RSBS-43 (**d**-**f**) derived from squamous cell carcinoma and adenocarcinoma respectively. RSBS-14- **a** High nucleus to cytoplasmic ratio, **b** Hemidesmosomes, **c** Keratin filaments. RSBS-43- **d** Prominent nucleolus, **e** Cell junctions **f** Prominent rough endoplasmic reticulum

Electron microscopy of the cell lines derived from squamous cell carcinoma (RSBS-9,-14 and − 23) showed high nucleo-cytoplasmic ratio with keratin filaments in the cytoplasm and cell junctions in the form of hemi-desmosomes. RSBS-43 cell line derived from adenocarcinoma cervix showed a few irregular microvilli, prominent nucleolus and prominent rough endoplasmic reticulum and occasional cell junctions. Representative images from RSBS-14 and RSBS-43 are shown in Fig. 2.

Karyotyping was done on all the four cell lines at passage 32. All 4 cell lines displayed aneuploidy. The number of chromosomes ranged from 47 to 53 in the RSBS-9, from 44 to 47 in RSBS-14, from 47 to 57 in RSBS-23 and from 47 to 53 in the RSBS-43 cell lines. The modal chromosome number was 53, 47, 56 and 51 in the RSBS-9, 14, − 23 and − 43 respectively [Additional file 2: Figure S1].

### Variable nucleotide tandem repeat assay and HPV typing

**Fig. 3:**
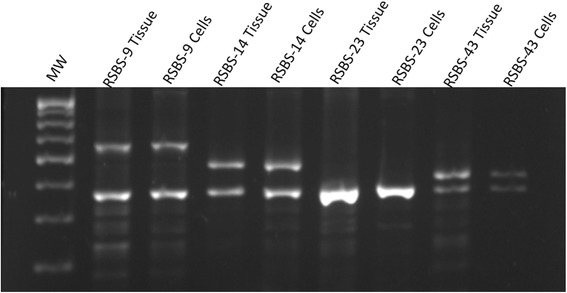
Variable Nucleotide Tandem Repeat Assay. Range of amplification product varies from 387 to 723 bps. The cell lines show a pattern identical to the respective parental tissue and which are unique to them

The VNTR assay uses multiple primer sets read as informative or non-informative. This established that the cell lines were indeed derived from the corresponding tissue sample and are unique [Fig. 3].

HPV DNA typing for HPV-16, − 18, − 31, and − 45, the most common HPV types in cervical cancer, revealed positivity only for HPV-16 sequence (NCBI Reference sequence NC_001526, nt31-nt683) [Additional file 3: Figure S2A].

### Cell growth kinetics, cell cycle analysis and cytotoxicity assay to determine cisplatin sensitivity

Cell cycle analysis showed 9–15% cells in the S-phase and 0.05–7.6% in subG1 phase [Additional file 3: Figure S2B]. The estimated doubling time was approximately 48 h for all the four cell lines [Additional file 3: Figure S2C, companion file].

Cisplatin is commonly used in cervical cancer as part of the chemotherapy protocol and hence cisplatin sensitivity was determined in these cell lines [Additional file 3: Figure S2D]. The IC-50 for RSBS-9 and RSBS-14 was 33 μM and 27 μM which are relatively more chemoresistant as compared to the IC50 for RSBS-23 and RSBS-43 which was 12 μM and 10 μM respectively.

### Tumorsphere formation, self-renewal and differentiation

Tumorsphere formation could be demonstrated from cells derived from the primary cultures obtained from biopsy specimen of invasive cervical carcinoma, from the four novel cell lines established and from HeLa and SiHa cell lines. In these cases, tumor cells were dissociated and grown under anchorage-independent or ‘stem-cell’ conditions Floating aggregates of cells formed small spheroids on day 7 and representative images (RSBS-14 and RSBS-43) are shown in Additional file 4: Figure S3A; further, the spheroids increased in size and contained 300–400 cells by day 14. Primary spheres were collected, mechanically disrupted to obtain a single cell suspension. The dissociated single sphere-forming cells were diluted and again grown under similar stemness conditions to obtain secondary spheroids and this cycle could be repeated establishing the self-renewal ability of the tumorspheres. Further, sphere-derived cells were dissociated and shown to grow in standard culture conditions as adherent monolayers indicating capability of differentiation.

### ALDH activity in cervical cancer derived cell lines

Adherent cultures from the four novel cell lines as well as SiHa and HeLa cell lines were tested by the ALDEFLUOR assay which identifies cancer stem cell population based on the high aldehyde dehydrogenase activity (ALDH) activity of cells in the presence or absence of the ALDH inhibitor DEAB. Further, these cells were sorted by flow cytometry based on high / low ALDH activity. Compared with control cells treated by the DEAB inhibitor, we observed 2.9% to 6% cells to be ALDH^high^ whereas rest were ALDH^low^ in Hela, SiHa and four novel cell lines. A representative plot is shown in Additional file 4: Figure S3B. This established that a subpopulation of cells in cervical cancer cell lines exhibit high ALDH activity. In order to confirm that these cells represent functional cancer stem cells, we characterized the tumorsphere-formation ability of ALDH^high^ and ALDH^low^ cells. Equal numbers of cells (15,000 cells) of ALDH^high^ and ALDH^low^ cells were cultured and tumorspheres was assessed at day 10. ALDH^high^ positive cells showed higher capacity for spheroid formation [Additional file 4: Figure S3B].

### Flow cytometric evaluation for expression of stem cell markers

Next, we wished to identify the surface phenotypic expression of putative stem cell markers in the tumorspheres enriched for cancer stem cells. Initially,the expression of known stem cell markers including CD44, CD49f, CD133 and CD71, CD90, CD105 was tested by flow cytometry in the tumorspheres and adherent bulk cells in each of the 4 cell lines RSBS-9, − 14, − 23 and − 43 and also HeLa and SiHa cell lines. CD71, CD90 and CD105 did not show any differential expression and so were not pursued further. There was a differential expression of CD44, CD49f and CD133. CD44 levels were higher in adherent cells as compared to tumorshpere cells. Adherent cells showed CD49f expression in the range of 45.7–89% whereas in tumorspheres it ranged from 61.1–90.7%. Similarly CD133 positivity ranged from 1.4 to 19% percentage in adherent cells whereas it was 12.5–63.6% in corresponding tumorspheres. A representative plot in RSBS-9 cell line is shown [Additional file 5: Figure S4]. Thus both CD49f and CD133 showed higher levels in tumorspheres than in adherent cells with a 2–5 fold difference in CD49f and a 3.3–7 fold difference in CD133 making them candidate CSC markers.

### Direct Immunofluroscence revealed expression of CD133 in tumorspheres

**Fig. 4:**
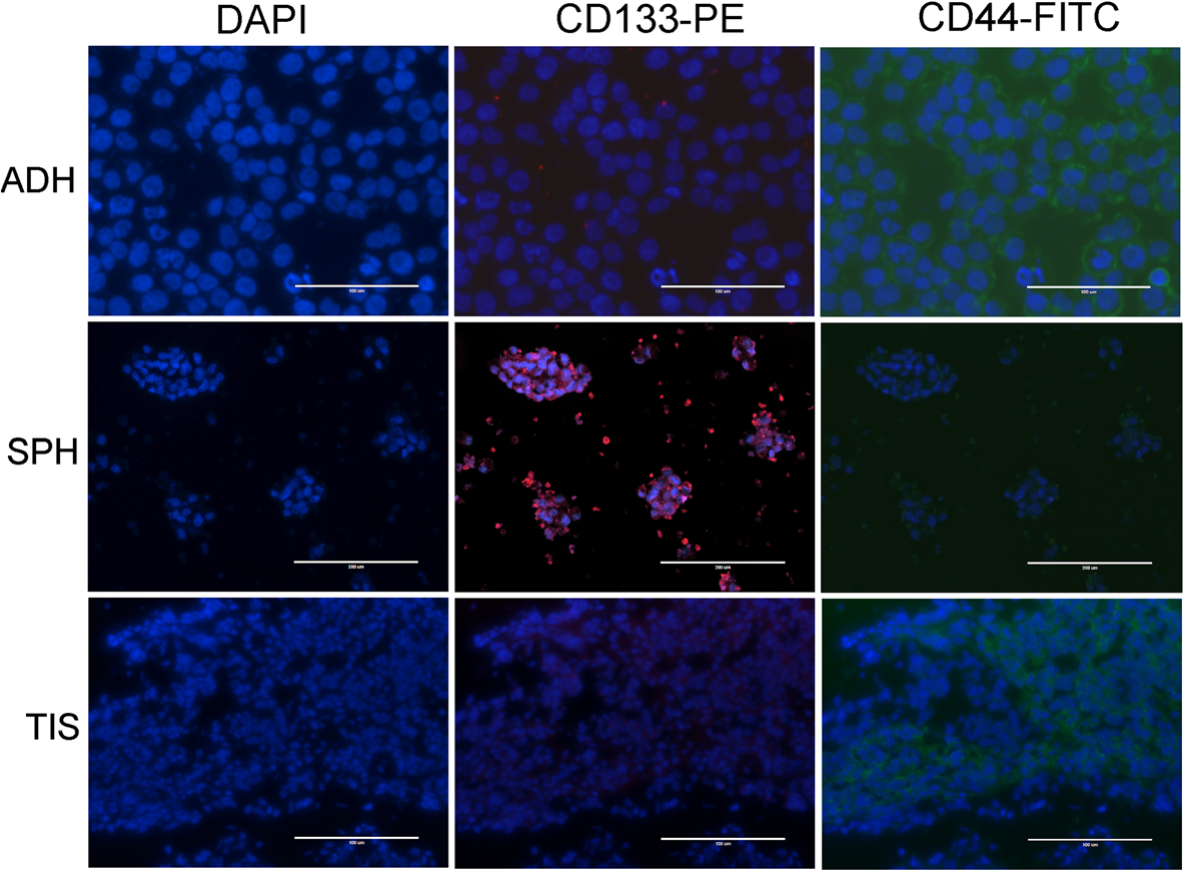
Direct immunofluorescence on adherent cells (ADH), tumorspheres (SPH), and tissue sections (TIS). Representative image panel of RSBS-14 cell line showing CD133 brightly positive in tumorspheres but not in adherent cells; whereas CD44 is positive in adherent cells and tissue sections but almost absent in tumorspheres

Direct immunofluorescence was carried out on adherent cells and tumorspheres derived from all four novel cell lines and their corresponding tissue sections. Similar results were observed in all cell lines tested and a representative image panel from RSBS-14 cell line is shown in Fig. 4. Adherent cells and tissue sections from invasive cervical cancer showed diffuse positivity for CD44 and were absent in tumorsphere derived cells. The levels of CD49f was similar to CD44 in adherent cells and weak expression in tumorspheres [Additional file 6: Figure S5]. On the other hand, only CD133 showed differential expression with high levels in the tumorspheres and almost nil expression in adherent cells and in the tissue section [Fig. 4]. These observations confirm that CD133^+^ is a surface phenotypic marker of cervical cancer stem cells.

### Analysis of cancer stem cell surface marker in tissue samples of patients with cervical cancer including early relapsed cases

**Fig. 5:**
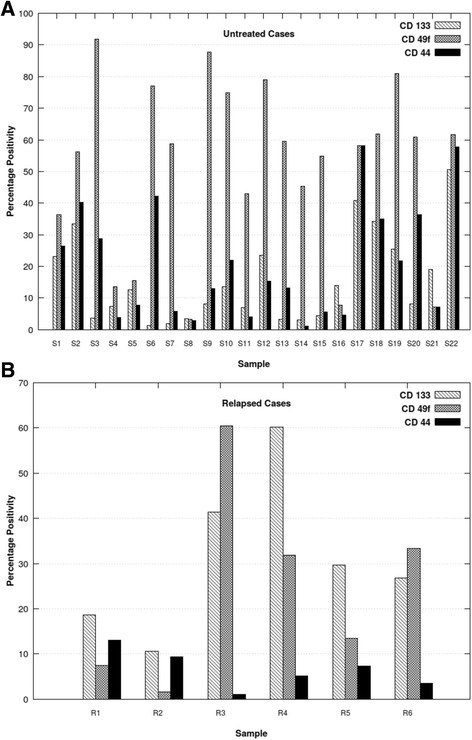
Flow cytometric immunophenotyping of invasive cervical cancer tissue biopsies for expression of CD133, CD49f and CD44 **a** Untreated cases. **b** Relapsed cases. Samples S1-S18 represent squamous cell carcinoma and S19-S22 represent adenocarcinoma. All relapsed cases were squamous cell carcinoma. CD133 levels are lower in untreated squamous cell carcinoma as compared to adenocarcinoma and also are lower than CD44 and CD49f levels. Relapsed cases showed upregulation of CD133 and downregulation of CD44 as compared to untreated cases

Expression of CD44, CD49f and CD133 in patient samples by flow cytometry are shown in Fig. 5a and Fig. 5b respectively. In untreated cases (*n* = 22), CD133 showed 1.3–50.6% positivity (median 10.4%); CD49f showed 3.2–91.8% positivity (median 58.4%), CD44 showed positivity in 1.2–58.1% (median 14.3%). In 6 cases who had received chemoradiation as per standard protocol but had relapsed cases within 1 year and hence represent radiation resistance or treatment failure, there was a trend towards downregulation of CD44 (median 6.2%) and upregulation of CD133 (median 28.2%). CD49f expression was very variable and no trend was observed.

### CD133^+^ cancer stem cells, tumorosphere forming abiltiy and response to cisplatin

**Fig. 6:**
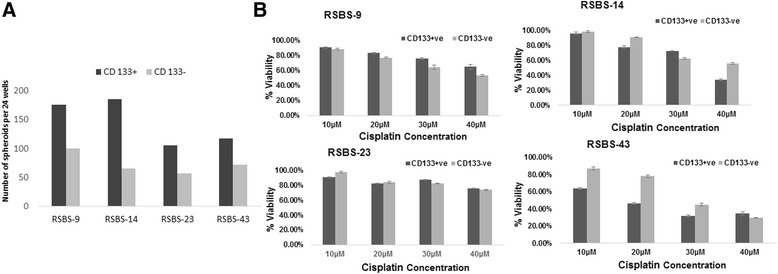
**a** Tumorsphere assay in sorted CD133^+^ vs CD133^−^ cells. Average numbers of tumorspheres are depicted. Note nearly 2–3-fold increased ability to form spheres in CD133^+^ cells. **b** Chemosensitivity assay to cisplatin in the four cells lines comparing CD133^+^ vs CD133^−^ cells. No appreciable differences in sensitivity was observed

CD133^+^ cancer stem cells were sorted from adherent cultures of RSBS 9, − 14, − 23 and − 43 cells tumorosphere formation assessed. CD133^+^ cells formed more and larger spheroids as compared to CD133^−^ cells [Fig. 6a]. Further, the response of CD133^+^ cancer stem cells to cisplatin determined by MTT assay. There was no appreciable difference in the CD133^+^ versus the CD133^−^ cells in the response to cisplatin in all 4 cell lines tested [Fig. 6b].

### Expression of stemness and epithelial mesenchymal transition specific transcripts in CD133^+^cancer stem cells

**Fig. 7:**
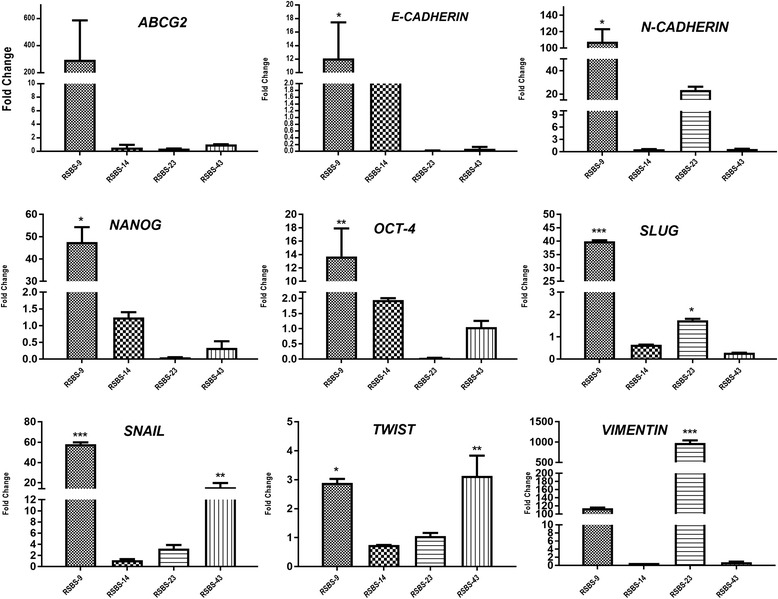
Quantitative real-time PCR for various Stemness and epithelial-mesenchymal transition markers in CD133^+^ sorted cells as compared to CD133^−^ cells (baseline) and expressed as fold change. Average values of experiments performed three times in duplicate is shown along with standard deviation Note variable upregulation of the different markers in the four cell lines. [ANOVA, * indicates *p* < 0.05, ** indicates *p* < 0.01 and *** indicates *p* < 0.001]

Relative expression of stemness markers (*OCT4, NANOG*) and EMT markers (*VIMENTIN, E-CADHERIN, N-CADHERIN, TWIST, SNAIL AND SLUG*) in CD133^+^ sorted cells in relation to unsorted cells and after normalization to *β-ACTIN* housekeeping gene was evaluated and results shown in Fig. 7. CD133^+^ RSBS-9 cells showed marked upregulation of *OCT-4, NANOG,* ABCG-2, *SLUG, SNAIL, TWIST, N CADHERIN,* and *VIMENTIN*; however, *E-CADHERIN* was also upregulated. CD133^+^RSBS-14 cells showed upregulation of *OCT-4* and *NANOG*, upregulated *E-CADHERIN* with little change in the other markers. CD133^+^ RSBS-23 cells showed upregulation of *VIMENTIN, TWIST, SNAIL, SLUG AND N-CADHERIN,* nil expression of *E-CADHERIN* with no change in the other markers. CD133^+^ RSBS-43 cells showed upregulation of *OCT-4, TWIST and SNAIL* and downregulation of *E-CADHERIN* with no appreciable change in the other transcripts. Taken together, our results indicate a variable albeit an overall upregulation of stemness and EMT markers in the CD133^+^ sorted cancer stem cells in all the four cell lines evaluated.

## Discussion

In this study, we established four cervical carcinoma derived cell lines from Indian subjects designated RSBS-9, RSBS -14, RSBS -23 representing squamous cell carcinoma and RSBS -43 representing adenocarcinoma, the two major types of invasive carcinoma of the uterine cervix. Each of these cell lines has unique modal chromosome numbers and have been proved to be derived from the parental tissue biopsy specimen as evidenced by identical band patterns in the VNTR assay. The overall success rate for establishing cell lines was 21.2% which compares favourably with a 26% success rate in a previous report on cell lines derived from invasive cervical cancer; however, the cells were grown on an irradiated layer of 3 T3 fibroblasts to achieve successful growth in the initial passages which was not required in the present study [9]. On the other hand, in gastric carcinoma, a very low success rate of just 3% from primary gastric tumors was reported although it was 25% from metastatic sites [10]. Differences in the histological characteristics of a tumour, especially the tumor microenvironment and associated features such as desmoplasia and inflammation could explain the differences in the success rate between the two sites. The role of prior tumor xenografts in athymic nude mouse models in improving success rates has been highlighted by a report on the development of colon cancer cell lines [11].

Out of the 6 commonly used cell lines in cervical cancer research, CaSki and HT-3 cell line are derived from metastatic sites of cancer the small intestine and lymph node respectively, whereas SiHa, HeLa, C-4 II and C-33A are derived from primary sites. The CaSki cell line was developed following surgery and irradiation. All the 4 cell lines we have developed are from primary cervical cancer who had not received any form of prior therapy.

All cell lines reported here grew as adherent monolayers in standard conditions and as tumorspheres under ‘stemness’ conditions successfully and hence these cell lines could serve as useful models for study of cancer stem cell biology as well. The morphology of the cell lines was in keeping with the parent tissue histology as evidenced in the cell blocks prepared from the cell lines. This was further corroborated by ultrastructure studies which revealed hemidesmosomes and keratin filaments in the squamous cell carcinoma derived cell lines in comparison to a prominent nucleolus, abundant rough endoplasmic reticulum and small surface microvilli in the adenocarcinoma derived cell line.

All the four cell lines were HPV16 positive which is the most frequent type of HPV associated with cervical carcinoma, both worldwide as well as in India [12–15]. In the meta-analysis of by Bhatla et al. [13], in invasive cervical carcinoma, HPV-16 was the most common type seen in 68.4% followed by HPV-18 in 14.7% cases; even in adenocarcinoma histotype, HPV-16 is more frequent (34.5%). Multiple infections from more than one type of HPV was seen in only 14% cases. Several studies have demonstrated the requirement of HPV oncogenic expression for proliferation, growth or survival of primary cervical cancer cells in culture [16–18]. Among the commonly used cervical cancer derived cell lines, CaSki and SiHa are positive for HPV-16, HeLa, C-41 are HPV-18 positive whereas C-33A and HT-3 test negative for HPV sequences.

The cell lines developed by us showed variable chemosensitivity to cisplatin with RSBS-23 and RSBS-43 relatively more chemosensitive and RSBS-9 and RSBS-14 more chemoresistant. In a previously published report, HeLa cell line is reported to be more chemosensitive as compared to SiHa which is cisplatin resistant [19]. Thus the variable chemosensitivity of these cell lines reflects better tumor biology and variation in the chemotherapeutic response and hence are useful in screening of therapeutic agents which are of potential value in invasive cervical cancer.

The next part of the study was aimed at isolation and characterization of the cancer stem cells using these low-passage cell lines. We employed two well-known assays which enrich for cancer stem cells. These included the ALDEFLUOR functional assay which enriches for ALDH^high^ cells [20], and the tumorsphere assay which too enriches for CSC [21]. The tumorsphere assay is considered as a surrogate assay for cancer stem cells and correlates well with in vivo tumorigenicity assay [4]. Feng et al. reported CD44 as a putative CSC marker based on its expression in sphere-derived cells, however, comparison of its level in non-tumorsphere cells was not performed. In the current study, CD44 was not upregulated in spheres; rather, the levels were lower than in adherent cells upon flow cytometry, further corroborated by direct immunofluorescence studies on adherent versus tumorspheres. Moreover, tissue sections of invasive cervical cancer expressed high levels of CD44 (by flow Cytometry and direct IF) making it highly unlikely to be a cancer stem cell marker. The two promising markers were CD49f and CD133. Lopez et al. used HeLa, SiHa, Ca Ski and C-41 cell lines to derive sphere-forming cells and showed CD49f expression at high levels (> 90% cells) and CD133 (1.4–61%) at lower levels and so concluded that CD49f is a putative marker [8]. We observed a 2–3-fold upregulation of CD49f in sphere-derived cells as compared to adherent cells; however, by direct immunofluorescence and flow cytometry, CD49f levels were high in the adherent cells and in tissue specimen and hence less likely to be a CSC marker. On the other hand, CD133 showed a 10–13-fold difference between adherent cells versus sphere-derived cells with low levels in the adherent cells upon flow cytometry and further confirmed by direct immunofluorescence studies. The CD133^+^ sorted cells also showed a greater ability to form spheres as compared to the CD133^−^ bulk cells providing further evidence in support of it being a marker of CSC. A limitation of this study is the lack of corroborative in vivo tumorigenicity studies in immunocompromised mice; however, the tumorsphere assay is considered as a surrogate assay [6] which was employed extensively in this study.

Screening of untreated cervical carcinoma tissue biopsies revealed median CD133 levels were 10.4% and this satisfies one of the defining criteria of stem cells that they should constitute a minority of the tumor cell population. We recruited 6 cases of carcinoma cervix who had relapsed following chemoradiation therapy to provide proof-of-principle for the involvement of cancer stem cells in radiation-resistance. Supporting this hypothesis, these relapsed cases showed upregulation of CD133 and down-regulation of CD44. Thus, our study provides in-vivo evidence for the role of CSC in radiation-resistance corroborating the in vitro evidence demonstrated previously in cell lines wherein sphere-derived cells were shown to be more resistant to radiation than their adherent counterparts [8].

Our observations that CD133+ is a phenotypic marker of cancer stem cells in carcinoma cervix are supported by a recent study by Tyagi et al. who have further shown the role of the HPV E6 oncogene in maintenance of the stemness characteristics of cancer stem cells in carcinoma cervix [22]. An overall but variable upregulation of the stemness and EMT related transcripts in the CD133^+^ CSC was observed confirming previous reports [4, 8]. Out of the 4 cell lines, RSBS-23 showed marked vimentin expression with concomitant low levels of E-cadherin implicating epithelial-mesenchymal transition occurring even in the basal state. The heterogeneity of the expression of stemness and EMT markers among the CD133^+^ cancer stem cells in these low-passage cell lines better reflects the in vivo picture rather than studies on highly passaged cell lines.

## Conclusion

Four new low-passage cervical cancer derived cell lines have been generated which will provide future researchers with a better model system to study tumor biology and cancer stem cells. CD133^+^ is a putative marker of cancer stem cells as it satisfies the defining criteria of ability for self-renewal, differentiation, restriction to a minority of the tumour and consistent expression enabling their isolation.

## Additional files

Additional file 1: (DOCX 20 kb)

Additional file 2: **Figure S1.** Representative karyotypes with modal chromosome numbers of RSBS-9 (A), RSBS-14 (B), RSBS-23 (C) and RSBS-43 (D) respectively. (TIFF 127 kb)

Additional file 3: **Figure S2. A.** PCR for HPV-16 in cell lines and corresponding parental tissues showing positivity for all cell lines and the parental tissues. **B.** Cell cycle analysis. **C.** Cell growth curve and estimation of doubling time showing similar doubling time of 48 h approximately. **D.** Cisplatin sensitivity assay showing variable chemosensitivity in the cell lines. (TIFF 192 kb)

Additional file 4: **Figure S3. A.** Representative figure of tumorspheres from RSBS-14 and RSBS-43 cell lines on day 7. **B.** Aldefluor assay: Representative plots of RSBS-14 cell line showing 3.2% cells with high ALDH levels. Cell sorted into ALDH low (−) and high (+) levels and tumorsphere assay performed showing spheres in ALDH^high^ sorted cells. (TIFF 223 kb)

Additional file 5: **Figure S4.** CD133 and CD49f expression in adherent (ADH) vs Tumorspheres (SPH) in RSBS-9 cell line by Flow Cytometric Immunophenotyping. Representative plots of CD49f and CD133 expression and data represented in histograms. Both markers showed increased expression in tumorspheres as compared to adherent cells. (TIFF 123 kb)

Additional file 6: **Figure S5.** CD49f expression in adherent cells and tumorspheres. Representative images panel of RSBS-14 cell line showing similar and moderate levels in adherent cells and in tumorspheres. (TIFF 2512 kb)

## Abbreviations

ADH: Adherent cells
ALDH: Aldehyde Dehydrogenase
APC: Allophycocyanin
CD: Cluster of Differentiation
CSC: Cancer stem cell
DAPI: 4′,6-diamidino-2-phenylindole
DNA: Deoxyribonucleic acid
EMT: Epithelial Mesenchymal Transition
FACS: Fluorescence-activated cell sorting
FITC: Fluorescein isothiocyanate
HPV: Human Papilloma Virus
MTT: 3-(4,5-Dimethylthiazol-2-Yl)-2,5-Diphenyltetrazolium Bromide
PE: Phycoerythrin
Per CP: Peridinin-chlorophyll-protein
PI: Propidium Iodide
RSBS: Cell lines named after the scientists
SPH: Spheroids
TIS: Tissue
VNTR: Variable Number Tandem Repeat
