# Supplementary material for: Significance of CD133 positive cells in four novel HPV-16 positive cervical cancer-derived cell lines and biopsies of invasive cervical cancer
Source: BMC Cancer. 2018 Apr 2;18:357. doi: 10.1186/s12885-018-4237-5 (PMC5879557; doi:10.1186/s12885-018-4237-5)
Supplement: Supplementary file 1 — (DOCX 20 kb) [file 12885_2018_4237_MOESM1_ESM.docx]

**Electron Microscopy**

Approximately 5x10^5^ cells from RSBS-14 and RSBS-43 cell lines were centrifuged gently and fixed in 3% glutaraldehyde in Sorensen’s phosphate buffer, pH 7.2 for 4-6 hours followed by secondary fixation in 1% Osmium tetroxide in Millonig’s phosphate buffer pH 7.2. The cell buttons were processed and embedded in Taab-812 embedding medium. Ultrathin sections of 60nm thickness were cut on Leica EM UC-6 ultramicrotome (Leica Mikrosysteme GmbH, Wien, Austria) and taken on Nickel grids. Ultrathin sections were stained with uranyl acetate and lead citrate and examined at 80kV accelerating voltage at JEOL Transmission Electron Microscope, JEM-1400Plus (JEOL, Tokyo, Japan) equipped with XR81M-B Camera (Advanced Microscopy Techniques Corp, Woburn, MA, United States). Digital electron micrographs were acquired using AMT Image Capture Engine V602 software (Advanced Microscopy Techniques Corp, Woburn, MA, United States).

**Karyotyping**

Karyotyping was performed on metaphase spreads of RSBS-9, RSBS-14, RSBS-23 and RSBS-43 cells at passage 35 by standard G-banding. Metaphase cells were collected after 3-hour colcemid (0.2 μg/mL) exposure and incubation in 0.075 M KCl solution at 37°C for 40 min. After fixing with a mixture of methanol and glacial acetic acid (3:1, v/v), cell suspensions were spread onto cold slides. The slides were stained with 3% Giemsa for morphological examination and chromosomes analysis. G-banding of the chromosomes was carried out by standard trypsin-Giemsa banding technique. Karyotypes were analysed in at least 10 cells in accordance with the International system for Human Cytogenetic Nomenclature.

**ALDEFLUOR Assay**

Adherent cells derived from samples were used for ALDEFLUOR assay using ALDEFLUOR kit (Stem cell Technologies) as per manufacturer’s instructions detailed in supporting information. 1 × 10^6^ cells in 1ml were taken in tubes labelled as test and control for each sample to which, 5μL of activated ALDEFLUOR substrate was added and 5 μL of DEAB solution (inhibitor) was added to only the control tube. The reagents and cells were mixed properly and incubated for 60 minutes at 37°C and were centrifuged for 5 mins at 250g. The cell pellets were resuspended in 0.5mL of ALDEFLUOR Assay Buffer and were analysed by Flow Cytometry acquiring at least 100,000 events per sample. Based on their ALDH activity, cells were sorted as ALDH^high^ and ALDH^low^ cells.

**Detection of putative markers of cancer stem cells by Flow Cytometry and FACS**

At least 1x10^6^ cells derived from tumorspheres and corresponding adherent cells were screened for surface markers by flow Cytometry using fluorescent (FITC, PE, APC, Per-CP) labelled antibodies against CD117 PE (BD biosciences USA), CD90 PE (BD biosciences USA, CD105 APC (Biolegend,USA), CD44 FITC (BD biosciences USA), CD49f PE(BD biosciences USA), CD 71 APC (Biolegend,USA) and CD133 PE (Miltenyi Biotech, Germany). Isolation of the CD133^+^ cells was performed using a FACS Aria flow cytometer (BD Biosciences, USA) and analyzed further.

**Direct Immunofluorescence**

Direct immunofluorescence was carried out on adherent cells and tumorspheres with FITC/PE labelled antibodies against CD44, CD49f and CD133 and on corresponding frozen tissue sample of cervical cancer (supporting information). 2000 cells derived from adherent cells and cervicospheres was dissolved with minimum amount of PBS and spun onto a slide using a Cytospin™ 4 Cytocentrifuge (Thermo scientific, USA) and frozen tissue sections obtained using a cryostat. The slides were fixed for 30 minutes in ice cold 100% methanol, dried and incubated with respective antibodies for 45 minutes at 37ºC in the dark. Nuclei were counter stained with DAPI. Florescence microscopy was carried out with EVOS FL Auto cell imaging system (Invitrogen, Thermo Fisher Scientific, USA).

**Screening of patients samples of invasive cervical carcinoma for cancer stem cell percentage and patients after radiotherapy**

Flow cytometric immunophenotyping (FCI) was performed on the biopsies of patients with untreated invasive cervical cancer (n=22) and in 6 patients who had been treated with chemoradiation therapy 46 Gy/23 fraction external beam radiation administered concurrently with 40 mg/m2 cisplatin weekly dose followed by intracavitary brachytherapy and who had subsequently relapsed. The levels of CD133, Cd49f and Cd44 was evaluated in each case after gating for CD45 negative cells supporting information). Briefly, 20 mg of tissue was minced mechanically by a scalpel, syringed thrice and filtered using a 70µm mesh to obtain a single cell suspension. Cells were incubated at 37ºC for 45 minutes with CD133 PE, CD49f PE and CD44 FITC labelled antibodies at room temperature. Negative control consisted of omission of antibody. The cells were acquired in the flow cytometer (BD FACS Aria, USA) and percent positivity evaluated.

**Human Papillomavirus (HPV) screening**

All cell lines developed were evaluated for HPV DNA type by PCR with type specific primers against HPV-16, -18, -31 and -45 (supporting information Table -2)

Table 1: Primers used for HPV DNA typing

| **HPV Type** | Primer | Annealing temperature |
| --- | --- | --- |
| ***HPV-16*** | F-CGT AAC CGA AAT CGG TTG AAC  R-GGACCATCTATTTCATCCTCCT | 63 ºC |
| ***HPV-18*** | F-CGGTGTATATAAAAGATG  R-TGCTCGTGACATAGAA | 56 ºC |
| ***HPV-31*** | F-GGAGTGACCGAAAGTGGTGAA  R-CTTGTCCAGCTGGACTGTCTA | 63 ºC |
| ***HPV-45*** | F-TGACGATCCAAAGCAACG  R- CCTACGTCTGCGAAGTCT | 62 ºC |

**MTT assay**

Cells from all the 4 cell lines generated and which were at the same passage 35 (P35) were used to plot the growth curve by using the MTT 3-(4,5-dimethylthiazol-2-yl)-2,5-diphenyl tetrazolium bromide assay detailed in supporting information. 5 × 10^3^ cells per well were plated into 96 well plates, and 7 plates per cell line. One plate of each cell line was assayed every 24 hours. The absorbance was measured in a microtiter plate reader at 570 nm as the test wavelength and 690 nm as the reference wavelength. The number of cells was calculated by the result of MTT compared with the cell number at the first day. The growth curves were plotted and the population doubling time of these 4 cell lines were calculated during the exponential growth phase of the cells.

**Cell cycle analysis**

Cell cycle analysis was performed after labelling the cellular deoxyribonucleic acid (DNA) with propidium iodide (PI) (Invitrogen, Carlsbad, CA, USA). After two cold PBS rinses, 5X10^4^cells were harvested during the exponential growth phase, fixed with cold 70% alcohol, and then incubated for 4 hours at 4^0^C. After centrifugation at 250g for 3 minutes and cold PBS rinse, cells were re-suspended in 1 mL propidium iodide stain (50 mMoL/L propidium iodide, 100 mg/mL RNase) and incubated for 30 min at room temperature. The cells were then subjected to cell cycle analysis by FACS (BD FACS Aria II). Each experiment was analysed in duplicate and four independent experiments were performed.

**Variable Nucleotide Tandem Repeat [VNTR] Assay**

Highly polymorphic VNTR loci D1S80 and IGH (VNTR at the immunoglobin heavy chain enhancer HSS 1.2) were used to prove and validate that the cervical cancer cell lines were derived from primary cervical cancer biopsies. Amplification of VNTR loci D1S80 (range of amplification product 387 to 723 bps) was achieved using the primers 5'-GAAACTGGCCTCCAAACACTGCCCGCCG- 3' (forward) and 5'-GTCTTGTTGGAGATGCACGTGCCCCTTGC-3' (reverse) and the primer sequence for IGH (range of amplification product 520 to 1720 bps) was 5’- GGGCCCTGTCTCAGCTGGGGA-3’ (forward) and 5’- TGGCCTGGCTGCCCTGAGCAG-3’ (reverse) with annealing temperature of 65ºC.

**Quantitative Real Time PCR (qRT-PCR) for stemness / EMT markers**

**Table 2: List of Primers used for qRT-PCR for stemness / EMT markers**

| **Gene transcript** | **Primer Sequence** | **Annealing temperature** |
| --- | --- | --- |
| ***β Actin*** | F-ACTCTTCCAGCCTTCCTTCC  R-CGTCATACTCCTGCTTGCTGC | 62 ºC |
| ***ABCG2*** | F-CTGAGATCCTGAGCCTTTGG  R-TGCCCATCACAACATCATCT | 60 ºC |
| ***OCT-4*** | F-ACATCAAAGCTCTGCAGAAAGAACT  R-CTGAATACCTTCCCAAATAGAACCC | 57.8ºC |
| ***NANOG*** | F-TTCCTTCCTCCATGGATCTG  R-TCTGCTGGAGGCTGAGGTAT | 56.1 ºC |
| ***E-CADHERIN*** | F-TGCCCAGAAAATGAAAAGG  R-GTGTATGTGGCAATGCGTTC | 56.8 ºC |
| ***SLUG*** | F-GGGGAGAAGCCTTTTTCTTG  R-TCCTCATGTTTGTGCAGGAG | 59.1ºC |
| ***VIMENTIN*** | F-GAGAACTTTGCCGTTGAAGC  R-GCTTCCTGTAGGTGGCAATC | 58.1 ºC |
| ***SNAIL*** | F-CCTCCCTGTCAGATGAGGAC  R-CCAGGCTGAGGTATTCCTTG | 59.4ºC |
| ***TWIST*** | F-GTCCGCAGTCTTACGAGGAG  R-CCAGCTTGAGGGTCTGAATC | 61.2 ºC |
| ***N-CADHERIN*** | F-TGTTTGACTATGAAGGCAGTGG  R-TCAGTCATCACCTCCACCAT | 61 ºC |
