# Supplementary figures and images for: Significance of CD133 positive cells in four novel HPV-16 positive cervical cancer-derived cell lines and biopsies of invasive cervical cancer
Source: BMC Cancer. 2018 Apr 2;18:357. doi: 10.1186/s12885-018-4237-5 (PMC5879557; doi:10.1186/s12885-018-4237-5)

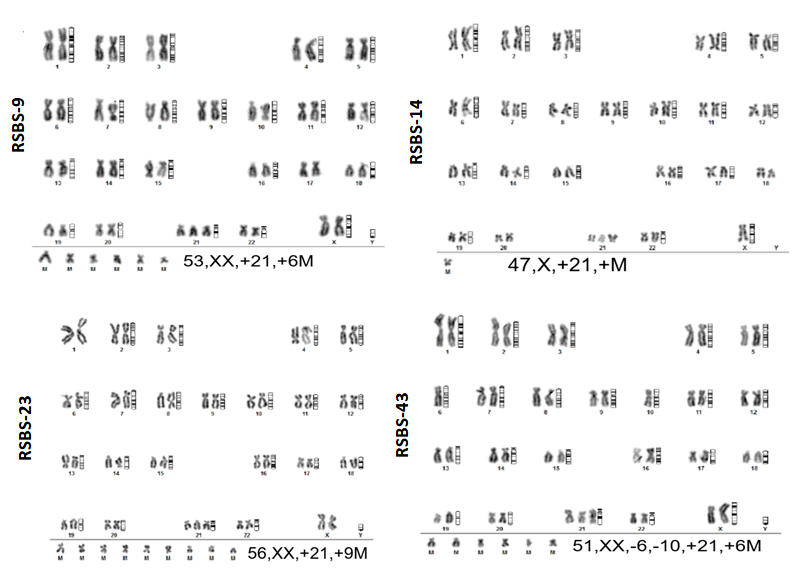

Supplement: Supplementary file 2 — Figure S1. Representative karyotypes with modal chromosome numbers of RSBS-9 (A), RSBS-14 (B), RSBS-23 (C) and RSBS-43 (D) respectively. (TIFF 127 kb) [file 12885_2018_4237_MOESM2_ESM.tif]

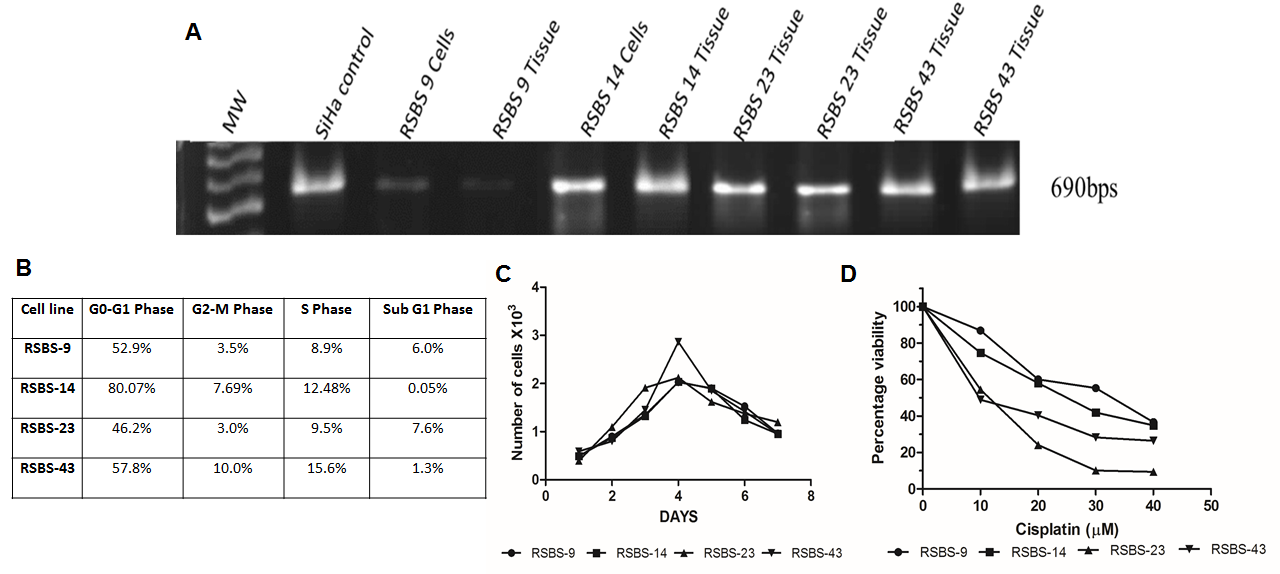

Supplement: Supplementary file 3 — Figure S2. A. PCR for HPV-16 in cell lines and corresponding parental tissues showing positivity for all cell lines and the parental tissues. B. Cell cycle analysis. C. Cell growth curve and estimation of doubling time showing similar doubling time of 48 h approximately. D. Cisplatin sensitivity assay showing variable chemosensitivity in the cell lines. (TIFF 192 kb) [file 12885_2018_4237_MOESM3_ESM.tif]

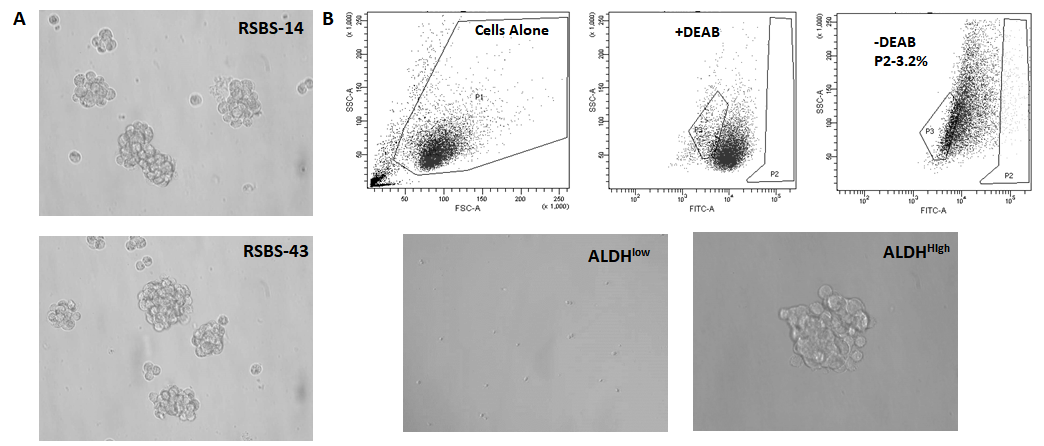

Supplement: Supplementary file 4 — Figure S3. A. Representative figure of tumorspheres from RSBS-14 and RSBS-43 cell lines on day 7. B. Aldefluor assay: Representative plots of RSBS-14 cell line showing 3.2% cells with high ALDH levels. Cell sorted into ALDH low (−) and high (+) levels and tumorsphere assay performed showing spheres in ALDHhigh sorted cells. (TIFF 223 kb) [file 12885_2018_4237_MOESM4_ESM.tif]

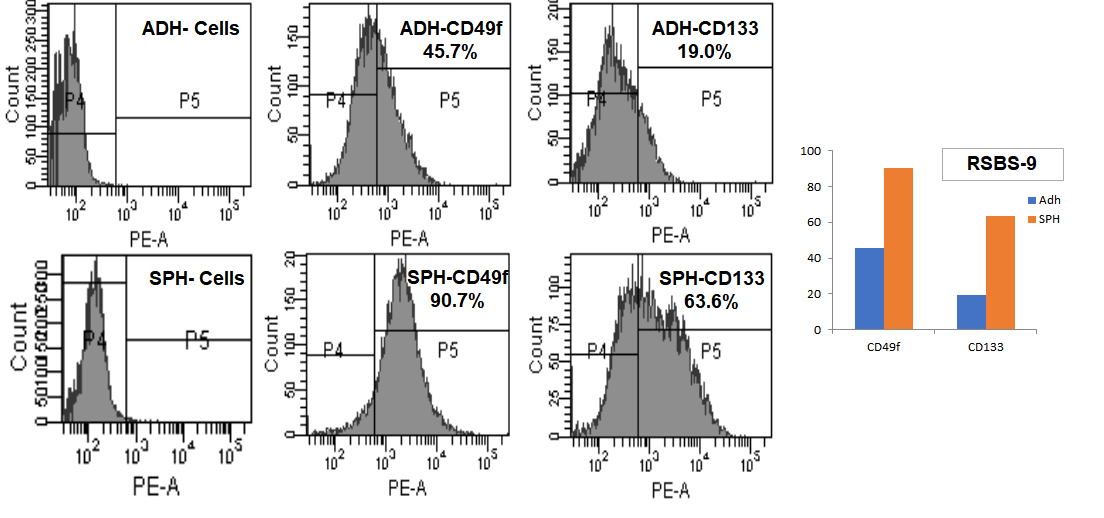

Supplement: Supplementary file 5 — Figure S4. CD133 and CD49f expression in adherent (ADH) vs Tumorspheres (SPH) in RSBS-9 cell line by Flow Cytometric Immunophenotyping. Representative plots of CD49f and CD133 expression and data represented in histograms. Both markers showed increased expression in tumorspheres as compared to adherent cells. (TIFF 123 kb) [file 12885_2018_4237_MOESM5_ESM.tif]

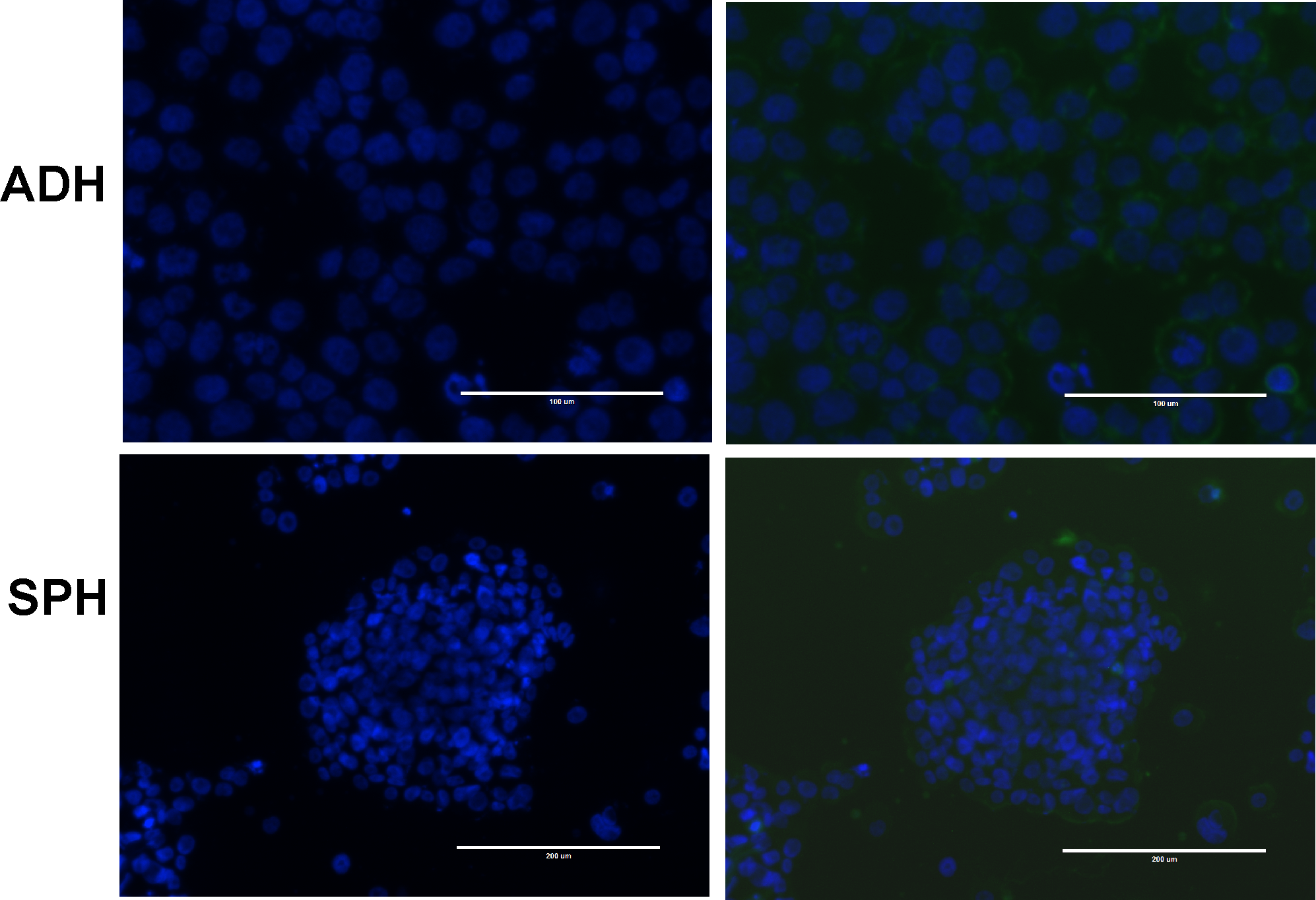

Supplement: Supplementary file 6 — Figure S5. CD49f expression in adherent cells and tumorspheres. Representative images panel of RSBS-14 cell line showing similar and moderate levels in adherent cells and in tumorspheres. (TIFF 2512 kb) [file 12885_2018_4237_MOESM6_ESM.tif]
